# Hypospermia Improvement in Dogs Fed on a Nutraceutical Diet

**DOI:** 10.1155/2018/9520204

**Published:** 2018-11-01

**Authors:** Francesco Ciribé, Riccardo Panzarella, Maria Carmela Pisu, Alessandro Di Cerbo, Gianandrea Guidetti, Sergio Canello

**Affiliations:** ^1^Ambulatorio Veterinario Città di Fermo, Via Falcone snc, Fermo, Italy; ^2^Ospedale Veterinario Himera, Via Antonio de Saliba 2, Palermo, Italy; ^3^Centro di Referenza Veterinario, Corso Francia 19, Torino, Italy; ^4^Department of Life Sciences, University of Modena and Reggio Emilia, Modena, Italy; ^5^Department of Medical, Oral, and Biotechnological Sciences, Dental School, University G. d'Annunzio of Chieti-Pescara, Chieti, Italy; ^6^Sanypet Spa, Research and Development Department, Bagnoli di Sopra, Padova, Italy; ^7^Research and Development Department, Forza10 USA Corp., Orlando, FL, USA

## Abstract

Male dog infertility may represent a serious concern in the canine breeding market. The aim of this clinical evaluation was to test the efficacy of a commercially available nutraceutical diet, enriched with* Lepidium meyenii, Tribulus terrestris, *L-carnitine, zinc, omega-3 (N-3) fatty acids, beta-carotene, vitamin E, and folic acid, in 28 male dogs suffering from infertility associated with hypospermia. All dogs received the diet over a period of 100 days. At the end of the evaluation period, no adverse effects, including head and tail anomalies percentage onset, were reported. Interestingly, motility percentage, semen volume and concentration, and total number of sperms per ejaculation significantly increased. Further investigations on a wider cohort of dogs might be useful to better correlate the presence of oxytetracycline in pet's diet and the onset of infertility and clearly assess the action mechanism of an oxytetracycline-free nutraceutical diet.

## 1. Introduction

Male dog infertility may result in important financial losses in the canine breeding market [1]. It can be due to the lack of or incomplete ejaculation or to poor semen quality [2]. While the first may occur if the coital lock is not adequate because of fear, anxiety, or discomfort during mating or at semen collection, the second can be due to poor quality of spermatozoa or abnormal seminal plasma (due to prostatic disease or to inflammation of the testis or of the epididymis).

Among predisposing factors of poor spermatozoa quality, there are congenital defects (testicular hypoplasia, the immotile cilia syndrome, and chromosomal abnormalities), cryptorchidism (the presence of only one or none testis in the scrotum) [1], duct system anomalies (cysts or developmental anomalies of the epididymis, the vas deferens, or the rete testis) [2], and prostate disorders (benign prostatic hypertrophy) (Ruel et al., 1998, prostatitis [3], prostatic cysts [4], and prostatic neoplasia [5]). Despite its poor characterization, also hypogonadism may be included in the aforementioned predisposing factors with a suspect familial tendency in some breeds [2].

The aim of this clinical evaluation was to test the efficacy of a commercially available nutraceutical diet in 28 male dogs suffering from infertility associated with hypospermia.

## 2. Materials and Methods

Twenty-eight, client-owned, male dogs, 3 Dogue De Bordeaux, 1 Bavarian Mountain Hound, 2 Jack Russell Terrier, 2 Boxer, 1 Czechoslovakian Wolfdog, 1 Pointer, 1 Basset Hound, 4 Bernese Mountain dog, 1 Leonberger, 1 Chihuahua, 2 French Bouledogue, 1 Shar-Pei, 1 Rottweiler, 1 Spanish Greyhound, 4 Bullmastiff, 1 Barbone Toy, and 1 St. Bernard (mean age ± SEM; 3.9 ± 0.3 years and mean weight ± SEM; 36.3 ± 3.5 Kg), suffering from infertility associated with hypospermia were enrolled in this clinical evaluation. Dogs received a nutraceutical diet over a period of 100 days.

All dogs received veterinary inspections, inclusive of prostate ecography in subjects with an age ≥ 4 years, before and at the end of the dietary regimen. Only dogs with a normal hematobiochemical profile were included in the evaluation.

At the beginning of the study (T0), after 60 days (T1), and after 100 days (T2), semen samples were collected from each dog and evaluated by means of a seminogram, which included spermatic volume, concentration, and motility, as well as the total number of spermatozoa per ejaculation and the percentage presence of tail and head anomalies.

The work was performed in compliance with national and international regulations (Italian regulation D.L. vo. 116/1992 and European Union regulation 86/609/EC) for procedures and animal care. The recommendations of the ARRIVE in animal research were also considered [6].

### 2.1. The Nutraceutical Diet

The diet, in the form of kibbles, was commercially available according to the Nutritional Guidelines of the European Pet Food Industry Federation [7]. It contained a standardized mixture of fish and rice and an omega-6:omega-3 ratio of 4:1 enriched with* Lepidium meyenii* (0.0865%)*, Tribulus terrestris *(0.0052%), L-carnitine (0.042%), zinc (0.005%), beta-carotene (0.023%), vitamin E (0.024%), and folic acid (0.000027%) embedded in cold-pressed tablets composed of 60-80% of hydrolyzed proteins (fish and vegetable).

### 2.2. Statistical Analysis

Data were analyzed using Prism 7 (GraphPad software, Inc., San Diego, USA). All data are presented as the means ± standard error of the mean and were first checked for normality using the D'Agostino-Pearson normality test. Differences in head and tail anomalies and motility percentage, as well as volume concentration and total count of sperms per ejaculation, at the beginning of the study (T0), after 60 days (T1), and after 100 days (T2) were analyzed using a one-way analysis of variance (ANOVA) followed by Tukey's multiple comparisons test. A value for ^*∗*^*p* < 0.05 was considered significant.

## 3. Results

**Figure 1:**
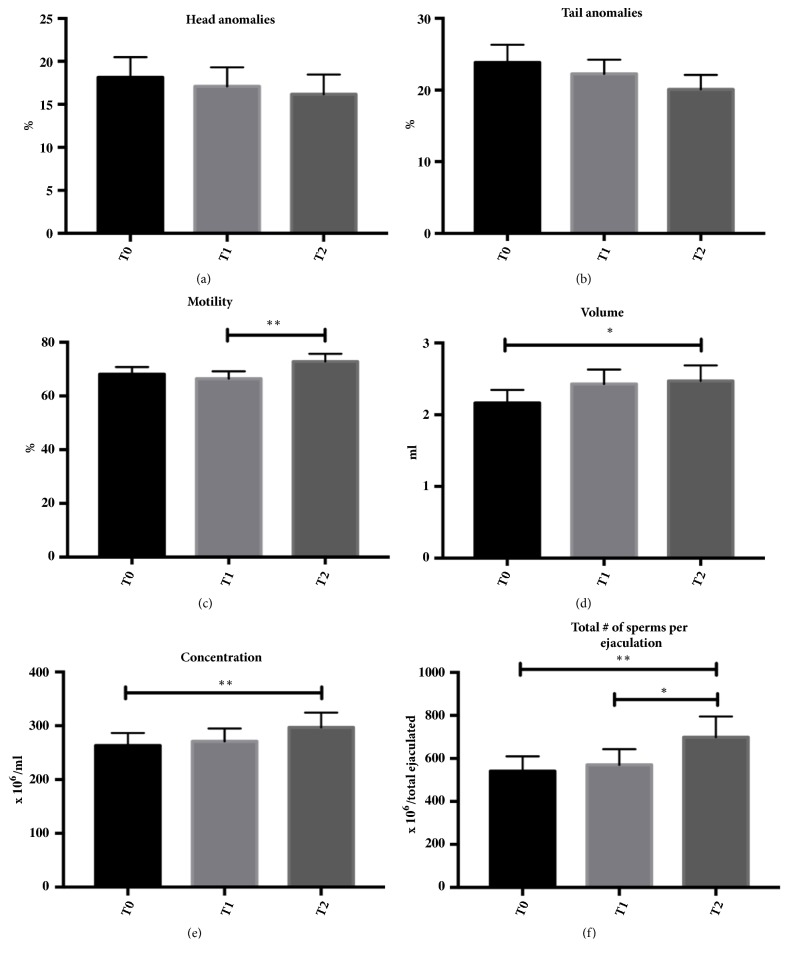
Graphical representation of spermatic volume, concentration, and motility, as well as the total number of sperms per ejaculation and the percentage presence of tail and head anomalies in dogs supplemented with the nutraceutical diet. *∗p* < 0.05; *∗∗p* < 0.01.

Twenty-eight dogs suffering from infertility associated with hypospermia were enrolled in the evaluation and received a nutraceutical diet enriched with* Lepidium meyenii, Tribulus terrestris, *L-carnitine, zinc, omega-3 (N-3) fatty acids, beta-carotene, vitamin E, and folic acid. No adverse effects, including head and tail anomalies percentage onset, were reported during the evaluation. In Figure 1, head and tail anomalies and motility percentage as well as volume concentration and total count of sperms per ejaculation at T0, T1, and T2 are shown.

Interestingly, motility percentage significantly increased from a T1 value of 66.43 ± 2.72 to 72.86 ± 2.85 at T2 (Figure 1(c)). Also semen volume reported a significant increase from a T0 value of 2.16 ± 0.18 ml to 2.47 ± 0.21 ml at T2 (Figure 1(d)). As to concentration, a significant increase from 263 ± 23.39 x10^6^/ml at T0 to 296.8 ± 27.73 x10^6^/ml at T2 was observed. Further, there was a significant increase in the total number of sperms per ejaculation from 541.5 ± 67.85 x10^6^/total ejaculated at T0 to 698.9 ± 96.27 x10^6^/total ejaculated at T2 and from 570.2 ± 72.58 x10^6^/total ejaculated at T1 to 698.9 ± 96.27 x10^6^/total ejaculated at T2.

## 4. Conclusions

Many literature reports investigated the relation between dietary intervention and fertility over the last decade [8–17].

This clinical evaluation aimed to investigate the efficacy of a commercially available nutraceutical diet in 28 male dogs suffering from infertility associated with hypospermia. Besides the significant increase in motility percentage, semen volume and concentration, and total number of sperms per ejaculation, these results are in agreement with those reported in literature where, for instance,* Lepidium meyenii *(maca) was used to enhance sexual desire [9, 10], improve mild erectile dysfunction [11] in men and quality of semen in animals [12, 13], and manage infertility [14]. The results of the latter also particularly improved by a pool of dietary supplements including L-carnitine, omega-3 (N-3) fatty acids, and a combination of zinc and folate [15]. On the contrary, an experimental reduction in the level of omega-3 (N-3) fatty acids negatively correlated with lower sperm and semen quality [16]. As to motility and spermatozoa increase, a similar trend was observed for human semen incubation with* Tribulus terrestris *[17].

Although diets favoring seafood, poultry, whole grains, fruits, and vegetables have been related to a better fertility in women and better semen quality in men [8], we recently highlighted the role of contaminants, e.g., oxytetracycline, dragged by food, e.g., chicken bone and meat meal, able to exert proinflammatory and cytotoxic effects* in vitro *[18–22] and* in vivo *[6, 23–27].

These previous observations led us to speculate that one of the triggering factors of testis or epididymis inflammation, which is the cause of a poor semen quality and in turn male dog infertility [2], might be due to the presence of oxytetracycline in commercially available pet food chronically and daily consumed by companion animals [23, 27].

Although we are aware that this evaluation would benefit of further* in vitro* investigations, including cytological serum evaluations for the presence of oxytetracycline to confirm our hypothesis, it also sheds light on a serious warning concerning the presence of toxic compounds, e.g., oxytetracycline, in pet food and their contribution to the etiopathogenesis of several clinical manifestations.

## Data Availability

The data used to support the findings of this study are included within the article.

## References

[1] Memon M. A. (2007). Common causes of male dog infertility. *Theriogenology*.

[2] Romagnoli S. (2006). Two common causes of infertility in the male dog. *World Small Animal Veterinary Association*.

[3] Smith J. (2008). Canine prostatic disease: A review of anatomy, pathology, diagnosis, and treatment. *Theriogenology*.

[4] Concannon P. W. (2000). *Recent Advances in Small Animal Reproduction*.

[5] Teske E., Naan E. C., Van Dijk E. M., Van Garderen E., Schalken J. A. (2002). Canine prostate carcinoma: Epidemiological evidence of an increased risk in castrated dogs. *Molecular and Cellular Endocrinology*.

[6] Kilkenny C., Browne W. J., Cuthill I. C., Emerson M., Altman D. G. (2012). Improving bioscience research reporting: the arrive guidelines for reporting animal research. *Veterinary Clinical Pathology*.

[7] FEDIAF http://www.fediaf.org/.

[8] Gaskins A. J., Chavarro J. E. (2018). Diet and fertility: a review. *American Journal of Obstetrics & Gynecology*.

[9] Gonzales G. F., Córdova A., Vega K. (2002). Effect of *Lepidium meyenii* (MACA) on sexual desire and its absent relationship with serum testosterone levels in adult healthy men. *Andrologia*.

[10] Stone M., Ibarra A., Roller M., Zangara A., Stevenson E. (2009). A pilot investigation into the effect of maca supplementation on physical activity and sexual desire in sportsmen. *Journal of Ethnopharmacology*.

[11] Zenico T., Cicero A. F. G., Valmorri L., Mercuriali M., Bercovich E. (2009). Subjective effects of *Lepidium meyenii* (Maca) extract on well-being and sexual performances in patients with mild erectile dysfunction: a randomized, double-blind clinical trial. *Andrologia*.

[12] Gonzales G. F., Gonzales-Castañeda C., Gasco M. (2013). A mixture of extracts from Peruvian plants (black maca and yacon) improves sperm count and reduced glycemia in mice with streptozotocin-induced diabetes. *Toxicology Mechanisms and Methods*.

[13] Clément C., Kneubühler J., Urwyler A., Witschi U., Kreuzer M. (2010). Effect of maca supplementation on bovine sperm quantity and quality followed over two spermatogenic cycles. *Theriogenology*.

[14] Canales M. (2000). Nutritional evaluation of Lepidium meyenii (MACA) in albino mice and their descendants. *Archivos Latinoamericanos de Nutrición*.

[15] Yao D. F., Mills J. N. (2016). Male infertility: Lifestyle factors and holistic, complementary, and alternative therapies. *Asian Journal of Andrology*.

[16] Rahman M. M., Gasparini C., Turchini G. M., Evans J. P. (2014). Experimental reduction in dietary omega-3 polyunsaturated fatty acids depresses sperm competitiveness. *Biology Letters*.

[17] Khaleghi S., Bakhtiari M., Asadmobini A., Esmaeili F. (2017). Tribulus terrestris Extract Improves Human Sperm Parameters In Vitro. *Evidence-Based Complementary and Alternative Medicine*.

[18] Di Cerbo A., Rubino V., Morelli F. (2018). Mechanical phenotyping of K562 cells by the Micropipette Aspiration Technique allows identifying mechanical changes induced by drugs. *Scientific Reports*.

[19] Gallo A., Landi R., Rubino V. (2017). Oxytetracycline induces DNA damage and epigenetic changes: a possible risk for human and animal health?. *PeerJ*.

[20] Guidetti G., Di Cerbo A., Giovazzino A., Rubino V., Palatucci A., Centenaro S. (2016). *In Vitro* Effects of Some Botanicals with Anti-Inflammatory and Antitoxic Activity. *Journal of Immunology Research*.

[21] Di Cerbo A., Palatucci A. T., Rubino V. (2016). Toxicological implications and inflammatory response in human lymphocytes challenged with oxytetracycline. *Journal of Biochemical and Molecular Toxicology*.

[22] Di Cerbo A., Scarano A., Pezzuto F. (2018). Oxytetracycline-Protein Complex: The Dark Side of Pet Food. *The Open Public Health Journal*.

[23] Di Cerbo A. (2018). Adverse food reactions in dogs due to antibiotic residues in pet food: a preliminary study. *Vet Ital*.

[24] Di Cerbo A., Sechi S., Canello S., Guidetti G., Fiore F., Cocco R. (2017). Behavioral Disturbances: An Innovative Approach to Monitor the Modulatory Effects of a Nutraceutical Diet. *Journal of Visualized Experiments*.

[25] Canello S., Centenaro S., Guidetti G. (2017). Nutraceutical approach for struvite uroliths management in cats. *International Journal of Applied Research in Veterinary Medicine*.

[26] Destefanis S. (2017). Clinical Evaluation of a Nutraceutical Diet as an Adjuvant to Pharmacological Treatment in Dogs Affected by Epiphora. *The International Journal of Applied Research in Veterinary Medicine*.

[27] Mazzeranghi F., Zanotti C., Di Cerbo A. (2017). Clinical efficacy of nutraceutical diet for cats with clinical signs of cutaneus adverse food reaction (CAFR). *Polish Journal of Veterinary Science*.

